# Primary Care Contact and Physical Health Monitoring in Severe Psychiatric Illness

**DOI:** 10.1192/bjo.2026.11130

**Published:** 2026-06-30

**Authors:** Émilie Smith, David Hayward, Donald MacIntyre, Douglas Steele

**Affiliations:** 1 University of Edinburgh, Edinburgh, United Kingdom; 2 NHS Lothian, Edinburgh, United Kingdom; 3 NHS Tayside, Dundee, United Kingdom

## Abstract

**Aims:**

Severe psychiatric illness (SPI), defined as schizophrenia and related psychoses, bipolar disorder (BPAD), and depression with psychosis, is associated with a reduced life expectancy of 15–20 years, largely attributable to physical illness.

SPI, and the sometimes-compulsory treatments, are associated with adverse effects on physical health, while other illness features like impaired decision-making ability, abnormal mood, and negative symptoms can impair engagement with health services. Consequently, regular monitoring and proactive management of physical illnesses are especially important. For these reasons, General Practice (GP) patient registers and outreach were previously considered necessary; however, the Quality and Outcomes Framework (QOF), which paid GPs to perform these roles, was withdrawn in Scotland in 2016 and cut back in England in 2025.

Therefore, we undertook a service evaluation of physical health monitoring in CMHT SPI outpatients. A constituent survey of contact with primary care services is reported here.

**Methods:**

CMHT keyworkers recorded BMI and asked their SPI outpatients when they last saw their GP and when physical health was last discussed in primary care.

**Results:**

Schizophrenia (n=212): 41% obese; schizoaffective disorder (n=11): 70% obese; BPAD (n=23): 56% obese; depression with psychosis (n=6): 80% overweight or obese; and delusional disorder (n=2). Among SPI patients with BMI ≥30, 47.1% had not seen their GP in over a year; for BMI ≥35, the figure was 46.5%; and for BMI ≥40, 45%. Overall, 51%(n=134) SPI patients thought they had seen their GP in the previous year, and only 36% (n=94) reported discussing physical health with a primary care provider during that time.

**Conclusion:**

32% of Scottish adults are obese, and 92% report seeing their GP at least annually, with contact rates higher among those with limiting long-term conditions. This contrasts with our SPI patient data, in which most were obese and only around half could recall seeing their GP within the past year, with only 36% recalling any discussion of physical health.

In 2012, SPI patients saw their GPs approximately three times annually. Our data suggest this level of engagement has declined markedly since then. As noted, the QOF incentives for GP management of the physical comorbidities associated with reduced life expectancy in SPI have been withdrawn in Scotland and cut back in England; this may have compounded an existing healthcare inequality and geographical disadvantage.

These data will be expanded to advocate for better community management of the physical health risks faced by people with SPI.

